# Characterization of Antimicrobial-Producing Beneficial Bacteria Isolated from Huanglongbing Escape Citrus Trees

**DOI:** 10.3389/fmicb.2017.02415

**Published:** 2017-12-07

**Authors:** Nadia Riera, Utpal Handique, Yunzeng Zhang, Megan M. Dewdney, Nian Wang

**Affiliations:** ^1^Citrus Research and Education Center, Department of Microbiology and Cell Science, Institute of Food and Agricultural Sciences, University of Florida, Lake Alfred, FL, United States; ^2^Citrus Research and Education Center, Department of Plant Pathology, Institute of Food and Agricultural Sciences, University of Florida, Lake Alfred, FL, United States

**Keywords:** plant growth-promoting bacteria (PGPB), citrus, microbiome, HLB, antimicrobial, antifungal compounds

## Abstract

The microbiome associated with crop plants has a strong impact on their health and productivity. *Candidatus* Liberibacter asiaticus (Las), the bacterial pathogen responsible for Huanglongbing (HLB) disease, lives inside the phloem of citrus plants including the root system. It has been suggested that Las negatively affects citrus microbiome. On the other hand, members of citrus microbiome also influence the interaction between Las and citrus. Here, we report the isolation and characterization of multiple putative beneficial bacteria from healthy citrus rhizosphere. Firstly, six bacterial strains showing antibacterial activity against two bacteria closely related to Las: *Agrobacterium tumefaciens* and *Sinorhizobium meliloti* were selected. Among them, *Burkholderia metallica* strain A53 and *Burkholderia territorii* strain A63 are within the β-proteobacteria class, whereas *Pseudomonas granadensis* strain 100 and *Pseudomonas geniculata* strain 95 are within the γ-proteobacteria class. Additionally, two gram-positive bacteria *Rhodococcus jialingiae* strain 108 and *Bacillus pumilus* strain 104 were also identified. Secondly, antimicrobial activity against three fungal pathogens: *Alternaria alternata*, *Colletotrichum acutatum*, *Phyllosticta citricarpa*, and two oomycetes: *Phytophthora nicotianae* and *Phytophthora palmivora.* Four bacterial strains *Burkholderia territorii* A63, *Burkholderia metallica* A53, *Pseudomonas geniculata* 95, and *Bacillus pumilus* 104 were shown to have antagonistic activity against the citrus root pathogen *Phytophthora nicotianae* based on dual culture antagonist assays and compartmentalized petri dish assays. The four selected bacteria were sequenced. Genes involved in phosphate solubilization, siderophore production and iron acquisition, volatile organic compound production, osmoprotection and osmotic tolerance, phytohormone production, antagonism, and nutrient competition were predicted and discussed related to the beneficial traits.

## Introduction

Citrus Huanglongbing (HLB, also known as citrus greening) is a devastating citrus disease (Bove et al., 1974; Wang et al., 2017a,b). HLB in Florida has been associated with the gram-negative α-proteobacteria *Candidatus* Liberibacter asiaticus (Las) which is fastidious and has not been cultured *in vitro* (Jagoueix et al., 1994; Bové, 2006). Las is transmitted to citrus plants mainly by the Asian citrus psyllid vector *Diaphorina citri* Kuwayama (do Carmo Teixeira et al., 2005). Once Las enters the phloem, it can multiply and spread throughout all phloem-containing tissues including leaf, bark, flowers, fruits, and roots (Tatineni et al., 2008). Las colonization and dispersion within the phloem is complex and it is suggested to depend on multiple environmental factors including temperature and solar radiation (Louzada et al., 2016).

Huanglongbing negatively affects the root system and the root-associated microbial community changes as the disease progresses. At late stages of the disease, HLB affects carbohydrate metabolism in the plant and changes root physiology by highly decreasing starch content (Etxeberria et al., 2009). It has been shown that Las titer is often first detected in the root system and significantly reduces root density even in trees that remain asymptomatic in aerial tissues (Johnson et al., 2014). In addition to the direct effect of Las on root physiology, HLB-affected root systems are predisposed to secondary infection with *Phytophthora nicotianae* which results in a greater damage to fibrous roots (Graham et al., 2013).

In the past decades, many studies have focused on understanding the role of the microbiome and its impact on plant health and productivity (Turner et al., 2013; Berg et al., 2014; Lebeis, 2014; Schlaeppi and Bulgarelli, 2014; Wagg et al., 2014). Some microbes in the rhizosphere of plants have been known to suppress diseases by competing for resources thereby making them unavailable for pathogens, promoting stress resistance and improving overall yield by providing nutrients (Lugtenberg and Kamilova, 2009; Mendes et al., 2011). The combination of these symbiotic microbes living in close contact with the root tissue and the plant itself is usually referred to as holobiont (Guerrero et al., 2013; Vandenkoornhuyse et al., 2015).

Interestingly, some healthy looking citrus trees have been identified in severely HLB-diseased citrus grove, and hereafter are referred to as HLB escape trees. Escape plants share the same genotype as symptomatic trees and are grown under similar environmental conditions. It has therefore been proposed that the HLB escape trees differ because of their associated microbial community composition (Sagaram et al., 2009). The microbial community of escape plants seems to be enriched in beneficial traits as compared to those of symptomatic trees (Trivedi et al., 2010). It has been hypothesized that the HLB escape trees might result from their microbiome via (1) antagonizing Las directly, (2) providing plant growth promotion factors, (3) antagonizing other pests present in the root zone, or (4) improving plant resistance to HLB or psyllids (Wang et al., 2017b). Here, we focus our attention in understanding the potential of putative beneficial bacteria in directly antagonizing Las, other citrus pathogens and promoting plant growth.

The goal of this study was to isolate and characterize antimicrobial-producing bacterial strains from the rhizosphere of HLB escape citrus trees. Additionally, the antimicrobial activity against other citrus pathogens was investigated. Furthermore, the production of volatile organic compounds (VOCs) was explored. Volatiles can serve either as antifungal compounds or as signaling molecules in the plant immune system (van Dam et al., 2016). To explore the genomic basis of identified beneficial traits, we also sequenced four bacterial isolates that showed promising beneficial traits. This study advanced our understanding of the roles of microbiome on HLB-escape trees and the potential application of beneficial bacterial in disease management.

## Materials and Methods

### Isolation of Bacteria from Healthy Citrus Rhizosphere

All bacterial strains used in this study were isolated from the rhizosphere of asymptomatic citrus trees in citrus groves with most trees showing severe HLB symptoms. One hundred and forty two isolates previously obtained from “Valencia” orange (*Citrus sinensis*) trees (Trivedi et al., 2011) were used in this study to screen for beneficial bacteria. Additionally, 200 bacterial isolates from “Cleopatra” mandarin (*C. reshni*) were collected from groves in Lake Wales, FL, United States. Trees in these selected groves were planted in 2003 and bacterial isolation was performed in 2013. Nutrient broth (NB), tryptic soy agar (TSA), and King’s B (KB) media were used for isolation as described previously (Trivedi et al., 2011).

### Bacterial and Fungal Growth Conditions

Unless otherwise noticed, all bacteria isolated from asymptomatic citrus rhizosphere were grown on nutrient agar (NA) plates or in NB with 180 rpm agitation and incubated at 28°C. *Sinorhizobium meliloti* and *Agrobacterium tumefaciens* were grown on LB and NA media, respectively. *Colletotrichum acutatum* and *Alternaria alternata* isolates were grown on potato dextrose agar (PDA) plates and incubated at room temperature for 7 days. *Phyllosticta citricarpa* was grown on half-strength PDA medium and incubated at 25°C with 12/12 h of light–dark photoperiods for 14–21 days. *Phytophthora nicotianae* and *Phytophthora palmivora* were grown on full-strength V8 medium or clarified V8 medium and incubated at room temperature for 7 days.

### Antibacterial Activity

Since Las has not been cultured to date, we used two bacteria (*S. meliloti* and *Agrobacterium tumefaciens*) that are closely related to Las as surrogates. These two species have been used as surrogates before to test antimicrobial activity against Las due to their close phylogenetic relationship with Las (Stover et al., 2013; Hu et al., 2016). Antibacterial activity was tested by inoculating the rhizospheric bacteria on NA plates and cross streaking the isolate in the center of the plate followed by incubation at 28°C. After 3 days, plates were subject to chloroform vapor overnight to kill the bacteria and were left in the fume hood until all the solvent has fully evaporated. A second layer of soft agar medium (0.6%) with an incorporated culture of *Agrobacterium tumefaciens* or *S. meliloti* at 10^2^ cfu/mL was set on top. The second layer was incubated at 28°C overnight. Positive inhibition was recorded when there was the presence of an inhibitory halo.

### Identification of Beneficial Bacteria and Phylogenetic Analysis

Identification of antimicrobial-producing strains was performed by amplifying 16s rRNA gene from selected bacteria using the universal primers 27F and 1492R (Lane, 1991). The corresponding amplification products were purified (Wizard^®^ SV Gel and PCR Clean-Up System) and sequenced with both primers and internal primer 519F. The sequences were assembled using Cap3 online software and further compared with the prokaryotic 16S rRNA gene sequence database EzTaxon (Huang and Madan, 1999; Kim et al., 2012). The 16S rRNA gene sequence of the closest type strain identified was recorded and the total 12 sequences were used for the phylogenetic tree construction. The 12 sequences were aligned using CLUSTALW algorithm and the phylogenetic tree was constructed using Neighbor Joining method with MEGA (Version 6.0) with a bootstrap value of 1000 (Saitou and Nei, 1987; Thompson et al., 1994; Tamura et al., 2013).

### Antimicrobial Activity against Fungi and Phytophthora spp. Pathogenic to Citrus

Many plant growth-promoting bacteria (PGPB) have the ability to inhibit fungal pathogens by producing antimicrobial compounds and/or extracellular enzymes. Here, we explored the ability of the selected antimicrobial-producing bacteria to inhibit growth of three fungi: *Alternaria alternata*, *Colletotrichum acutatum*, *Phyllosticta citricarpa*, and two oomycetes: *Phytophthora nicotianae* and *Phytophthora palmivora*.

Dual culture antagonist assays were performed as described previously (Fokkema, 1978). Briefly, the appropriate media for each species was used and a mycelial plug was placed in the center of the plate. One microliter of the bacterial suspensions at 10^8^ cfu/mL was inoculated 3 cm away from the mycelial plug in three sections of the plate.

The antimicrobial activity of volatile compounds was assessed using compartmentalized petri dishes as described previously (Fernando and Linderman, 1995). NA medium was used in one compartment for bacterial growth and clarified V8 medium was used for *Phytophthora nicotianae* mycelium growth. Each assay contained five replicates and the experiment was performed three times. Growth was monitored at 5 days after treatment and the *Phytophthora nicotianae* colony diameter was quantified using a ruler.

Statistical analysis of volatile-mediated mycelium inhibition was conducted using RStudio (Version 0.98.1049 – ©2009–2013 RStudio, Inc.) by applying Dunn’s test (package: dunn.test) with a *P*-value of 0.05 or less.

### Characterization of Beneficial Traits *in Vitro*

Screening for bacterial ability to solubilize phosphate was performed by plating each isolate in Pikovskaya medium (Pikovskaya, 1948). Strains showing a translucent halo surrounding the colony were considered positive for phosphate solubilization. Similarly, siderophore production was determined *in vitro* using CAS medium and monitored for the presence of a halo after incubation at 28°C for 24 h (Schwyn and Neilands, 1987).

### DNA Isolation and Genome Sequencing, Assembly, and Annotation

*Burkholderia metallica* strain A53, *Burkholderia territorii* strain A63, *Bacillus pumilus* strain 104, and *Pseudomonas geniculata* strain 95 were grown overnight in 1 mL of NB medium at 28°C with 180 rpm agitation. The cultures were centrifuged at 6,000 × *g* for 5 min and DNA was extracted using the Wizard^®^ Genomic DNA Purification Kit following the manufacturer’s instructions. DNA quality and quantity were measured using a ND-8000 NanoDrop Spectrophotometer (NanoDrop Technologies, Wilmington, DE, United States). Paired end reads (150 bp) were generated using an Illumina Hiseq2000 platform by Novogene Corporation for *Bacillus pumilus* strain 104 and *P. geniculata* strain 95. *Burkholderia territorii* strain A63 and *Burkholderia metallica* strain A53 were sequenced by BGI, Shenzhen using Illumina Hiseq2000 and paired end reads (125 bp) were generated. The reads were *de novo* assembled using CLC Genomics (version 8.0) for strains A63 and A53, with an iterative adaptive assembly approach, and the assemblies from k-mer 33 for *Burkholderia territorii* strain A63 and 29 for *Burkholderia metallica* strain A53 were chosen for further analyses for their highest reads utilization according to their longest average contig length. The *de novo* assembly was performed using MegaHit (version 1.06) for *Bacillus pumilus* strain 104 and *P. geniculata* strain 95. For each genome, functional annotation was completed using the RAST server (Aziz et al., 2008). Briefly, RAST is an online service that allows to predict genes encoding proteins as well as rRNA, tRNA genes in a given genome sequence. It will also classify the genes according to their function that can be used for further analyses (Aziz et al., 2008). The nucleotide sequence of the four bacterial strains and their annotations were submitted to the National Center for Biotechnology Information (NCBI) database.

### Identification of Genes Involved in Beneficial Traits and Antimicrobial Production

Protein family sorter tool from the Pathosystems Resource Integration Center (PATRIC) database was used to find relevant genes within the bacterial genomes (Meyer et al., 2009; Wattam et al., 2014). Families involved in phosphate solubilization, phytohormone production, synthesis of VOCs, and production of osmolytes and siderophores were searched in all genomes using the PATRIC visualizing tool. Prediction of the putative antimicrobial biosynthesis clusters was performed using antiSMASH software (version 3.0.5.) (Weber et al., 2015).

## Results

### Isolation of Antimicrobial Producing Bacteria and Bacteria Identification

**FIGURE 1:**
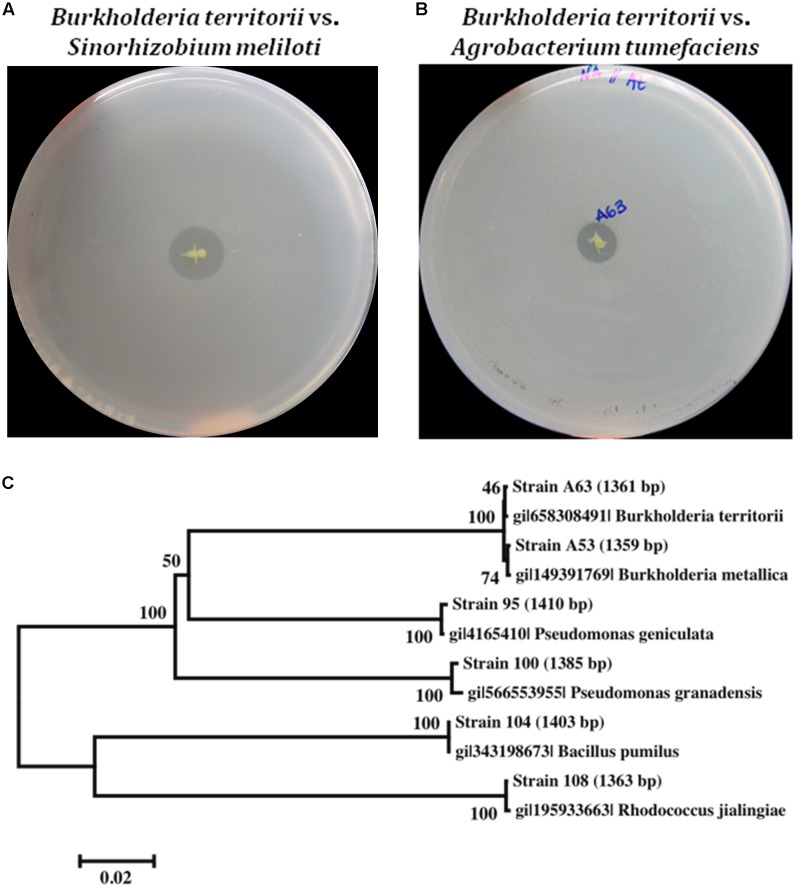
Antagonistic activity of selected beneficial bacteria. **(A)** Antagonist activity *in vitro* against the alpha proteobacteria *Sinorhizobium meliloti*. **(B)** Antagonist activity *in vitro* against the alpha proteobacteria *Agrobacterium tumefaciens*. **(C)** Phylogenetic tree of antibiotic-producing beneficial bacteria based on 16S rDNA sequences from the six beneficial bacteria and its closest type strain. Sequences were aligned using CLUSTALW algorithm and the phylogenetic trees were constructed using Neighbor Joining method with MEGA (Version 6.0).

In total, 342 isolates were screened for antimicrobial activity against *S. meliloti* and *Agrobacterium tumefaciens*, which are closely related to Las (Duan et al., 2009). Six isolates were observed to produce a growth inhibition zone, indicating antibacterial production, and were selected for further analysis (**Figures 1A,B**).

Identification of the antibiotic-producing bacteria was performed by amplifying the 16s rRNA gene. All amplicons obtained were 1350 bp or longer and were analyzed using the prokaryotic 16S rRNA gene sequence database EzTaxon (Kim et al., 2012). The closest type strain reported with the highest score was collected for further phylogenetic analysis (Supplementary Table 1). In total, 12 sequences were recorded and the resulting alignment was used to construct a phylogenetic tree (**Figure 1C**). Four of the six strains belong to the proteobacteria phylum, the most abundant phylum in the citrus microbiome (Zhang et al., 2017), with *Burkholderia metallica* strain A53 and *Burkholderia territorii* strain A63 within the β-proteobacteria class, whereas *Pseudomonas granadensis* strain 100 and *Pseudomonas geniculata* strain 95 within the γ-proteobacteria class. Additionally, two gram-positive bacteria were identified: *Rhodococcus jialingiae* strain 108 and *Bacillus pumilus* strain 104 belonging to the Actinobacteria and Firmicutes phyla, respectively. These six selected strains were further characterized for other beneficial features such as siderophore production and phosphate solubilization (Supplementary Table 2). Notably, *Burkholderia metallica* strain A53 was able to solubilize phosphate and *P. granadensi*s strain 100 was positive for siderophore production.

### Antimicrobial Activity against Phytophthora spp. and Citrus Pathogenic Fungi

It has been reported that Las infection in the root could make fibrous roots predisposed to *P. nicotianae* secondary infections (Graham et al., 2013). HLB may have more severe effect on fibrous root health when *P. nicotianae* is present (Wang et al., 2017b). Although the mechanisms behind this interaction are yet to be fully understood, one possible reason is that there is a higher attraction of *P. nicotianae* zoospores to Las infected roots (Wang et al., 2017b). In the competitive and complex root system, rhizospheric bacteria capable of inhibiting zoospores germination or hyphae growth may provide the plant with a competitive advantage to protect itself against *P. nicotianae* infection. Therefore, it is interesting to understand the antimicrobial activity for all six strains against *P. nicotianae* and *P. palmivora*, which cause root rot (both species) and foot rot (*P. nicotianae* only). We also evaluated their antifungal activity against three citrus pathogenic fungi, *Alternaria alternata*, *Colletotrichum acutatum*, and *Phyllosticta citricarpa*, which belong to the phylum Ascomycote and affect aerial tissues of the tree.

**FIGURE 2:**
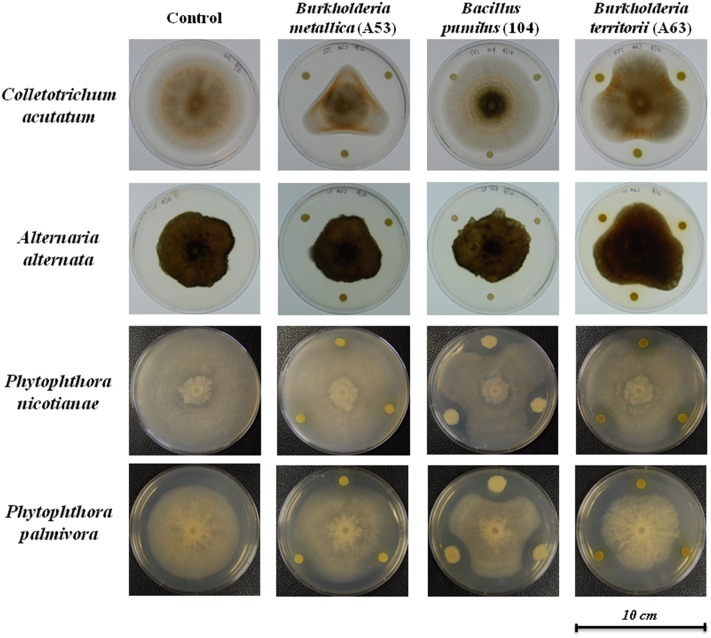
Dual culture antimicrobial activity *in vitro*. Activity of three antimicrobial producing bacteria was tested against *Colletotrichum acutatum*, *Alternaria alternata*, *Phytophthora nicotianae*, and *Phytophthora palmivora* by co-plating both organisms in culture media following incubation at room temperature for approximately 10 days. Inhibition was observed for *Burkholderia* strains against *C. acutatum*, *A. alternata*, and *Phytophthora*, and *Bacillus pumilus* against *Phytophthora*.

In dual culture antagonist assays, we observed that *Bacillus pumilus* strain 104 significantly inhibited the growth of *P. nicotianae* and *P. palmivora* (**Figure 2**), whereas *Burkholderia* spp. strains A53 and A63 only slightly inhibited their growth. Both *Burkholderia metallica* strain A53 and *Burkholderia territorii* strain A63 exhibited strong *in vitro* antifungal activity against *A. alternata* and *C. acutatum*. Consistently, both strains were able to inhibit fungal growth of *Phyllosticta citricarpa* and *C. acutatum* in an overlay assay (Supplementary Figures 1A,B). Interestingly, *Bacillus pumilus* strain 104 and both *Burkholderia* spp. strains were able to inhibit *Phytophthora nicotianae* growth in overlay assays with zoospores suggesting that they may inhibit spore germination (Supplementary Figure 1C). Similar results were observed with *Bacillus pumilus* strain 104, which completely inhibited fungal growth when challenged against *P. citricarpa* in the overlay assay (Supplementary Figure 1B).

**FIGURE 3:**
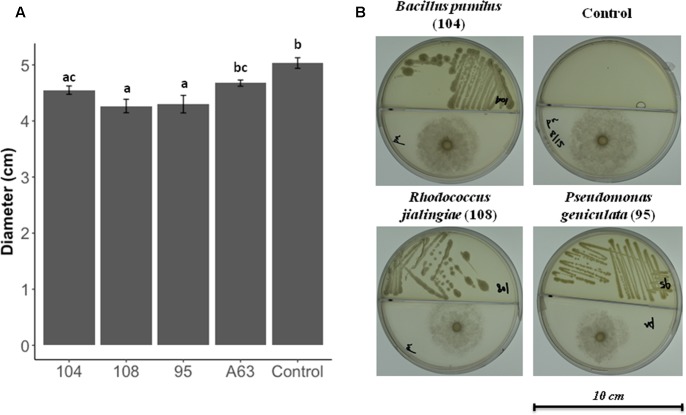
Antimicrobial activity against *Phytophthora nicotianae in* compartmentalized petri dishes **(A)** *Phytophthora nicotianae* growth in the presence or four antimicrobial producing beneficial bacteria. Error bars represent the standard error of the mean (*n* = 3). The experiment was repeated for three times (*n* = 15). Letters indicate significant difference based on Dunn’s test as compared to control [package: dunn.test, RStudio (Version 0.98.1049 – ©2009–2013 RStudio, Inc.)] with a *P*-value of 0.05 or less. *P*-values for all treatments are listed in Supplementary Table 3. **(B)** Pictures of *Phytophthora nicotianae* growth in the presence or antimicrobial producing beneficial bacteria.

Dual culture compartmentalized petri dishes were used to explore the possible antimicrobial activity by volatiles. *Bacillus pumilus* strain 104, *R. jialingiae* strain 108, and *P. geniculata* strain 95 were able to significantly reduce mycelial growth of *Phytophthora nicotianae* in compartmentalized cultures (**Figure 3**).

### General Features of the Genomes

The draft genomes of the four bacterial strains were obtained by using Illumina HiSeq2000 technology and *de novo* assembly. General information for the four draft genome sequences is summarized in **Table 1**. The mean coverage was 319× for *Bacillus pumilus* in 33 contigs. The GC content for *Bacillus pumilus* was 41.9% and the total genome size 3.7 Mb. RAST annotation identified 3894 features in this genome. The mean coverage was 411× for *P. geniculata* in 294 contigs. *P. geniculata* draft genome has a GC content of 65.8% and a genome size of 5.1 Mb. *Burkholderia territorii* (8.9 Mb) and *Burkholderia metallica* (8.2 Mb) draft genomes have a mean coverage of 105× and 112×, respectively. RAST annotation identified 8366, 7722, and 4615 features in *Burkholderia territorii*, *Burkholderia metallica*, and *P. geniculata* draft genomes, respectively.

**Table 1:** General features of the four draft genomes.

Strain name	NCBI accession number	Mean coverage	Number of contigs	Number of features^∗^	GC content (%)	Size (bp)
*Burkholderia territorii* strain A63	MUZF00000000	105×	341	8366	66.4	8,865,752
*Burkholderia metallica* strain A53	MULQ00000000	112×	347	7722	66.6	8,233,033
*Pseudomonas geniculata* strain 95	MULR00000000	411×	294	4615	65.8	5,080,605
*Bacillus pumilus* strain 104	MULS00000000	319×	33	3894	41.9	3,685,367


*^∗^Features include gene, CDS, rRNA, tRNA, misc RNA, and pseudogene.*

### Genes Responsible for Beneficial Traits

**FIGURE 4:**
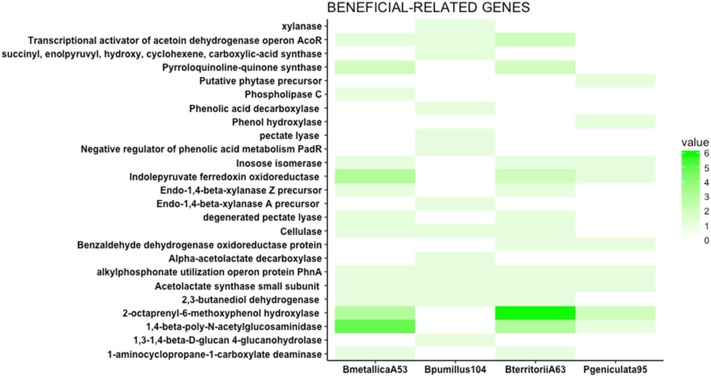
Beneficial traits related genes. The heat map represents the number of genes putatively involved in direct antagonism, phosphate solubilization, and nutrient facilitation. Phospholipase C, involved in the solubilization of phosphate, is only present in *Burkholderia metallica* strain A53. ACC deaminase (1-aminocyclopropane-1-carboxylate deaminase), an enzyme known to modulate ethylene levels in plants, is present in *Burkholderia metallica* strain A53 and *Burkholderia territorii* strain A63. 1,3-1,4-Beta-D-glucan 4-glucanohydrolase, cellulase, pectate lyase, xylanase, and endo-1,4-beta-xylanase A precursor identified in *Bacillus pumilus* strain 104 play roles in nutrient competition and fungal antagonism.

**FIGURE 5:**
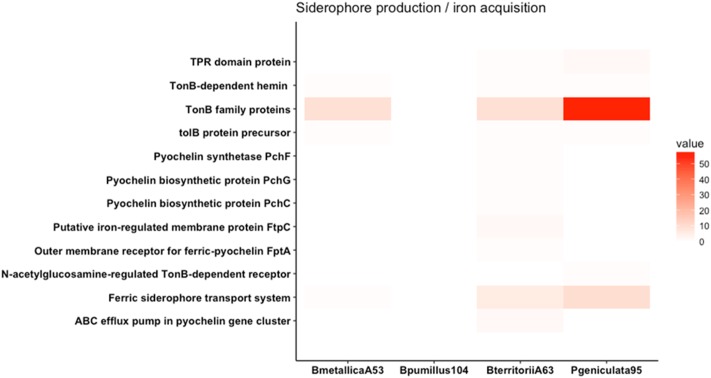
Heat map of genes involved in siderophore production and iron acquisition. Genes involved in pyochelin biosynthesis and TonB-dependant receptor family proteins are represented. *P. geniculata* strain 95 contains more than 50 TBDR family proteins.

The PATRIC database was used to search for genes typically involved in beneficial traits for the four strains. Namely, genes reported to be involved in phosphate solubilization, siderophore production and iron acquisition, VOC production, osmoprotection and osmotic tolerance, phytohormone production, antagonism, and nutrient competition were searched within the four genomes (**Figures 4**, **5** and Supplementary Figure 2).

#### Phosphate Solubilization and Iron Acquisition

*Burkholderia metallica* strain A53 was capable of phosphate solubilization (Supplementary Table 2). In the genomic content, it harbors a phospholipase C that may account for the *in vitro* phosphate solubilization activity (**Figure 4**).

Genes involved in siderophore production and iron acquisition are presented in **Figure 5**. Even though *P. geniculata* strain 95 does not produce siderophore in CAS media, it harbors more than 50 genes coding for TonB-dependent receptors (TBDR). TBDR are specialized receptors that transport siderophore-iron complexes in the periplasm of gram-negative bacteria (Cornelis, 2013). The presence of these receptors allows bacteria to sequester iron complexes produced from other organisms (Hartney et al., 2013). The overrepresentation of this feature in the genomic sequence has also been described in other relevant beneficial rhizobacteria from *Pseudomonas* and *Stenotrophomonas* genera (Berg et al., 2013; Cornelis, 2013).

#### Osmoprotection and Osmotic Tolerance

Many beneficial rhizospheric bacteria produce osmolytes that do not interfere with cellular functions, are highly soluble and can serve as osmoprotectors in drought stress conditions (Berg et al., 2013). Key players in this protection are trehalose and glucosylglycerol and some of the genes known to be involved in their biosynthesis are listed in Supplementary Figure 2. Alpha, alpha-trehalose-phosphate synthase, and trehalose-6-phosphate phosphatase are two enzymes important for the synthesis of trehalose, and both genes are present in *P. geniculata* strain 95 and the two *Burkholderia* strains A53 and A63.

#### Phytohormone Production

1-Aminocyclopropane-1-carboxylate deaminase (ACC deaminase) is known to regulate plant development by reducing levels of the plant hormone ethylene (Glick, 2005). ACC deaminase was found in the genomes of *B. metallica* strain A53 and *B. territorii* strain A63 (**Figure 4**). ACC deaminase is widely present in the *Burkholderia* genus (Onofre-Lemus et al., 2009).

The promoting activity of some plant growth-promoting bacteria is due to their ability to produce indole acetic acid (IAA), a phytohormone important for plant growth. The six antimicrobial-producing strains selected in this study were neither able to produce IAA nor to promote seed germination of tomato, corn, or soybean (data not shown). IAA production in bacteria involves at least three different pathways. Two of the best-characterized pathways use tryptophan as substrate: the indole-3-acetamide (IAM) pathway and the indole-3-pyruvate pathway. Additionally, there is a tryptophan-independent pathway. Consistent with our experimental data, the four sequenced bacteria lack most genes for IAA synthesis. However, indolepyruvate ferredoxin oxidoreductase and indole-3-glycerol phosphate synthase were found in the two *Burkholderia* strains and in *P. geniculata* strain 95. The former gene is found in Archaea. The latter gene has been associated with IAA biosynthesis by the tryptophan-independent pathway in *Arabidopsis thaliana* (Ouyang et al., 2000; Devi et al., 2015).

#### Antagonism and Nutrient Competition

Apart from the production of metabolites and phytohormones, an important role of the beneficial bacteria in the rhizosphere is the competition for nutrients, and their role in organic matter decomposition (Kamilova et al., 2005). Some enzymes such as cellulases, proteases, and glucanases are important for carbon cycling as well as antagonism of fungal pathogens (Gopalakrishnan et al., 2014). Therefore, the presence of glucanases, cellulases, and pectinases was investigated in the four genomes. Endo-1,4-beta-xylanase Z precursor, cellulase, and degenerated pectate lyase were found in the two *Burkholderia* strains. Similarly, 1,3-1,4-beta-D-glucan 4-glucanohydrolase, cellulase, pectate lyase, xylanase, and endo-1,4-beta-xylanase A precursor were identified in *Bacillus pumilus* strain 104 (**Figure 4**). Apart from their role in competition for nutrients, some of these enzymes may have a role in antagonizing fungal or oomycete pathogens because they can degrade cell walls. For instance, the presence of cellulase gene in *Bacillus pumilus* strain 104, *Burkholderia metallica* strain A53, and *Burkholderia territorii* strain A63 could potentially explain the antagonistic activity against *P. nicotianae in vitro* that was not seen for *P. geniculata* strain 95 (**Figure 2**).

#### Enzymes Involved in VOC Production

*In vitro* antagonism assay results suggest that *Bacillus pumilus* strain 104, *P. geniculata* strain 95, and *R. jialingiae* strain 108 produce VOCs that inhibit *Phytophthora* mycelium growth. *Bacillus* is known to produce acetoin and 2,3-butanediol as byproducts of incomplete oxidation of pyruvate and α-acetolactate (Effmert et al., 2012). The genomic sequence of *B. pumilus* strain 104 harbors the gene for 2,3-butanediol dehydrogenase (EC 1.1.1.4) that catalyses the production of 2,3-butanediol from 2-acetoin and the reverse reaction as well. This enzyme is also present in *Burkholderia metallica* strain A53 and *Burkholderia territorii* strain A63. Additionally, the gene that encodes the acetolactate synthase small subunit (EC 2.2.1.6) is present in all four strains. *Bacillus pumilus* strain 104 is the only one of the four strains that contains the alpha-acetolactate decarboxylase (EC 4.1.1.5) important for the synthesis of 2-acetoin from acetolactate (Supplementary Figure 3).

Some rhizospheric bacteria taxonomically related to *P. geniculata* strain 95 are able to produce VOCs. Namely, it has been reported that some *Stenotrophomonas maltophilia* strains isolated from soil, where tobacco was grown, were able to produce benzaldehyde, phenylacetaldehyde, and phenol as part of their active VOCs (Gu et al., 2007; Zou et al., 2007). These compounds were found to have both nematicidal as well as fungicidal activities. In *P. geniculata* strain 95 a putative benzaldehyde dehydrogenase (EC 1.2.1.28) was identified which catalyzes the production of methyl benzoate. Methyl benzoate is an active volatile that has been reported in other bacteria such as *Streptomyces* spp. and *Stigmatella* spp. (Schulz and Dickschat, 2007).

### Antimicrobial Biosynthesis Gene Clusters

A genome mining approach using AntiSMASH (version 3.0) tool was used to identify putative antibiotic biosynthesis gene clusters that could potentially explain the observed antimicrobial activity. The software predicted 10 clusters for *B. pumilus* strain 104, 9 for *P. geniculata* strain 95, and 15 clusters for each of the *Burkholderia* sp. strains (Supplementary Table 4).

*Bacillus pumilus* strain 104 contains two clusters of non-ribosomal peptide synthetase (NRPS) genes and one NRPS-PK hybrid gene cluster. One NRPS cluster identified had 95% gene similarity to the lychensyn biosynthetic gene cluster from *B. pumilus* strain 7P and is highly conserved within *Bacillus*. In addition, AntiSMASH software predicted a possible chemical structure for the identified lipopeptide (LP) (Supplementary Figure 4). Another NRPS cluster shares 53% gene similarity with the bacillibactin biosynthetic gene cluster. The NRPS-PK hybrid gene cluster has 85% gene similarity with the bacilysin biosynthetic gene cluster, a metabolite that has strong antibacterial effect against *Erwinia amylovora* and *Xanthomonas oryzae*, two phytopathogenic γ-proteobacteria (Chen et al., 2009; Wu et al., 2015). We also identified microcin, bacteriocin, and terpene gene clusters.

A total of nine gene clusters responsible for antimicrobial production were identified in the genome of *P. geniculata* strain 95. Among them, two clusters encode lantipeptides, one for lassopeptide, two for arylpolyene, two for bacteriocin, one for microcin, and one for NRPS. One lantipeptide gene cluster includes a lanthionine synthase C family gene and two leader/core peptide genes. This gene cluster also contains two regulatory genes: a sensor histidine kinase gene and a LuxR family DNA-binding response regulator gene downstream of the biosynthetic genes. For the second lantibiotic cluster no structure genes was identified. Lantibiotics are peptidic antimicrobial compounds produced in the ribosomes that undergo numerous post-transcriptional modifications. Lantibiotics were known to be produced by many gram-positive bacteria but lately they have been associated with other groups (Knerr and van der Donk, 2012). The lantibiotics predicted in *P. geniculata* strain 95 belongs to the class II of lantibiotics, produced by LanM-like proteins. LanM-like proteins are bifunctional proteins that assess the maturation of the leader peptide and the cyclation activity. According to *in silico* studies now it is known that *lanM*-like genes are wide spread in bacteria (Knerr and van der Donk, 2012).

In each *Burkholderia* strain, 15 clusters were identified to encode NRPSs, bacteriocin, PKS, terpene, phenazine, arylpolyene, phosphonate, etoin, and Hserlactone. Notably, both strains harbor the complete pyrrolnitrin biosynthesis cluster. Pyrrolnitrin is a well-characterized compound produced by many *Pseudomonas* and *Burkholderia* strains with strong antifungal and antibacterial activity (Hwang et al., 2002).

## Discussion

The positive interactions between the microbiome and citrus have been proposed as one of the possible reasons for HLB escape trees to delay HLB symptom development (Sagaram et al., 2009; Trivedi et al., 2010). The extended genome of the microbes associated with the escape plants may be responsible for a notable fitness advantage. This study aimed to identify potential antagonists of Las and characterize their beneficial activities in the rhizosphere that could assist a citrus tree to withstand infection by Las. One explored mechanism was if the antimicrobials produced by the citrus root holobiont suppressed the activity of bacterial and fungal citrus root pathogens. In addition, the water-soluble antibiotics produced by the rhizosphere microbial community might be absorbed into the xylem, which could further move into the phloem. It has been reported that in all vascular plants, phloem and xylem tissues are located next to each other, and there is clear evidence that these tissues exchange water (Sevanto, 2014). Six isolates belonging to Firmicutes, Actinobacteria, Betaproteobacteria, and Gammaproteobacteria with strong antimicrobial activity against *A. tumefaciens* and *S. meliloti* were identified. *A. tumefaciens* and *S. meliloti*, which are closely related to Las, have been used as surrogates because Las has not been cultured *in vitro* (Stover et al., 2013; Hu et al., 2016). Among the antimicrobial-producing isolates, *Burkholderia metallica* A53 and *Burkholderia territorii* strain A63, which were isolated from a mandarin rhizosphere, belong to the *Burkholderia cepacia* complex (BCC). BCC is a group of bacteria commonly found in the soil that are currently not allowed to be used in agricultural applications due to their potential risk to human health (Depoorter et al., 2016). It remains to be determined whether *Burkholderia metallica* strain A53 and *Burkholderia territorii* strain A63 can cause human diseases. Both *Burkholderia metallica* A53 and *Burkholderia territorii* strain A63 have the ability to modulate citrus immune system under greenhouse conditions when applied as soil drench. In addition, the *Burkholderiaceae* family was found to be a key taxa in citrus microbiome of healthy trees compared to that of HLB-symptomatic trees in the field (Zhang et al., 2017). Understanding the beneficial traits of *Burkholderia metallica* A53 and *Burkholderia territorii* strain A63 might provide useful hints on promoting citrus growth and disease management.

Antimicrobials produced by PGPB might also suppress eukaryotic plant pathogens. In Florida citrus groves, phytophthora foot and root rot are the most important soil-borne diseases of citrus with *Phytophthora nicotianae* being the most prevalent *Phytophthora* spp. in Florida citrus groves and nurseries (Colburn and Graham, 2007). *Burkholderia metallica* strain A53 and *Burkholderia territorii* strain A63 showed antimicrobial activity against *Phytophthora nicotianae*, *Phytophthora palmivora*, *A. alternata*, *C. acutatum*, and *Phyllosticta citricarpa*. It suggests that PGPB associated with HLB escape trees might be able to suppress fungal and oomycete pathogens. Bioinformatic analysis predicted the presence of the whole gene cluster for the production of pyrrolnitrin, a strong antifungal and antibacterial compound produced by not only *Burkholderia*, but also *Pseudomonas*, *Myxococcus*, *Serratia*, and *Enterobacter* spp. (el-Banna and Winkelmann, 1998). *Bacillus pumilus* strain 104 was able to inhibit *P. citricarpa* growth. Inhibition of spore germination by *Bacillus* spp. has been previously reported in *Bacillus pumilus* MSH against *Mucor* and *Aspergillus* spp. and in *Bacillus subtilis* YM 10-20 against *Penicillium roqueforti* (Bottone and Peluso, 2003; Chitarra et al., 2003). AntiSMASH prediction found that *B. pumilus* strain 104 contains a gene cluster encoding non-ribosomal peptide synthase (NRPS) class of antibiotics. One of the predicted NRPS is lichensyn a cyclic LP. An LP of the surfactin family has been reported to be involved in antifungal and antimicrobial interactions as well as facilitating root colonization and modulating plant immunity (Ongena and Jacques, 2008). Further characterization of the antifungal and anti-oomycota compounds of *Burkholderia* spp. might have broad impact on not only agricultural, but also medical fields.

Volatile organic compounds are lipophilic compounds with high vapor pressure, and low molecular mass that have an important role in antagonism, signaling, and cross-kingdom interactions in the rhizosphere. The chemical properties of VOCs allow them diffuse through water as well as air filled pores in the rhizosphere and in that way they can “connect” species that are physically separated (Kai et al., 2009; Effmert et al., 2012; Garbeva et al., 2014; Schmidt et al., 2015; Schulz-Bohm et al., 2016). Most microorganisms can produce VOCs as byproducts of their primary and secondary metabolisms. Many VOCs are produced from the oxidation of glucose (Korpi et al., 2009), and other VOC biosynthetic pathways include heterotrophic carbon metabolism, terpenoid biosynthesis, and fatty acid degradation among others (Peñuelas et al., 2014). VOCs are known to play two major roles in the rhizosphere: (1) as the chemical signals shaping the behavior and population dynamics of other microorganisms, and (2) as antimicrobial compounds suppressing or killing other microorganisms (Tyc et al., 2016). In this study, we addressed the possible antagonistic role of VOCs by evaluating the anti-oomycota activity *in vitro*. Inhibition of *P. nicotianae* growth by VOCs was significant for *R. jialingiae* strain 108, *P. geniculata* strain 95, and *B. pumilus* strain 104. It is noticeable that inhibition of *P. nicotianae* by *Bacillus pumilus* strain 104 may result from the production of VOCs or a combination of VOCs and antimicrobial compounds. Among the three strains with VOC producing activity, *B. pumilus* strain 104 contains all genes necessary for the production of 2,3-butanediol, a volatile that is usually produced by *Bacillus* strains and has important implications in plant immunity (Ryu et al., 2003; Rudrappa et al., 2010). Some VOCs are commonly produced among a group of bacteria, but frequently some strains produce unique specific types of VOCs (Schulz and Dickschat, 2007; Garbeva et al., 2014). Interestingly, 2,3-butanediol application has been used in the field due to its low production cost and low active concentration (Effmert et al., 2012; Piechulla and Degenhardt, 2014).

In Florida, *Phytophthora nicotianae* is recommended to be managed with metalaxyl applications rotated with phosphite salts because of the risk of resistant isolates (Graham and Feichtenberger, 2015). Recently, it has been proposed that these chemicals may be losing effect due to their interaction with HLB (Wang et al., 2017b). Interestingly, many bacterial strains are capable of suppression of *Phytophthora* spp. growth (Nakayama et al., 1999; Tran et al., 2007; Kim et al., 2008; Lee et al., 2008). Our isolates with strong capacity in inhibiting *Phytophthora nicotianae* have a potential in controlling *Phytophthora nicotianae*.

## Conclusion

Ensuring root health is critical to maintain a healthy citrus tree (Graham et al., 2013). Within the complex ecological interactions in the root system the microbiome plays an important role in sustaining the plant productivity. Although Las is not a soil-borne pathogenic bacteria, it has a strong impact in root health and their associated microbiome. It has been shown that the impact in the root systems occurs early in the plant–pathogen interaction before symptoms in the canopy begin to appear (Johnson et al., 2014). In addition, Las infection has increased root predisposition to *Phytophthora nicotianae* infection resulting in even further damage to the root health. Thus, rhizospheric bacteria capable of antagonizing Las or *P. nicotianae* could protect the root system in the early stages of Las infection. Here, we have isolated and characterized six beneficial bacteria with antimicrobial activity against two bacterial species taxonomically related to Las and the oomycete *Phytophthora nicotianae*. We have further investigated the genomic basis for the beneficial traits. Our previous study and ongoing study with application of few beneficial bacteria seem to suggest that manipulation of the soil microbes has no significant effect on HLB disease control once the infected trees become severely symptomatic. The beneficial microbes seem to slow down, but do not prevent Las infection when applied on healthy, asymptomatic trees, or symptomatic trees at the early stage of infection (Wang et al., 2017b), which is consistent with the nature of delayed HLB symptom development of HLB escape trees. It remains to be determined whether optimization of microbiome manipulation has a significant effect on HLB disease development.

## Author Contributions

NR and NW wrote the manuscript. NR and UH performed the experiments. NR and YZ did the genome sequence analysis. MD involved in experiments related to Phytophthora and fungi. All authors read and approved the manuscript.

## Conflict of Interest Statement

The authors declare that the research was conducted in the absence of any commercial or financial relationships that could be construed as a potential conflict of interest.
