# Supplementary material for: Characterization of Antimicrobial-Producing Beneficial Bacteria Isolated from Huanglongbing Escape Citrus Trees
Source: Front Microbiol. 2017 Dec 7;8:2415. doi: 10.3389/fmicb.2017.02415 (PMC5770638; doi:10.3389/fmicb.2017.02415)
Supplement: Supplementary file 1 [file Data_Sheet_1.docx]

Supplementary Material

Characterization of antibiotic-producing beneficial bacteria isolated from Huanglongbing escape citrus trees

Nadia Riera*, Utpal Handique, Yunzeng Zhang, Megan M. Dewdney, Nian Wang^1*^

*** Correspondence:** Nian Wang: [nianwang@ufl.edu](mailto:nianwang@ufl.edu)

# Supplementary Tables

Supplementary Table 1 Molecular identification of six antibacterial-producing strains.

| **Strain name** | **Isolation source** | **Isolation date** | **Antibacterial to *A. tumefaciens*** | **Antibacterial to *S. meliloti*** | **Sequence length (bp)** | **Closest type strain species** | **Closest Strain** | **Authors** | **Similarity (%)** |
| --- | --- | --- | --- | --- | --- | --- | --- | --- | --- |
| ***Pseudomonas geniculata* strain 95** | **'Valencia' orange** | **2010** | **+** | **+** | **1410** | ***Pseudomonas geniculata*** | **ATCC 19374** | **(Wright 1895) Chester 1901** | **99.86** |
| ***Pseudomonas granadensis* strain 100** | **'Valencia' orange** | **2010** | **+** | **+** | **1385** | ***Pseudomonas granadensis*** | **F-278,770(T)** | **Pascual et al. 2015** | **99.63** |
| ***Burkholderia territorii* strain A63** | **'Cleopatra' mandarin** | **2013** | **+** | **+** | **1361** | ***Burkholderia territorii*** | **LMG 28158(T)** | **De Smet et al. (in press)** | **99.93** |
| ***Burkholderia metallica* strain A53** | **'Cleopatra' mandarin** | **2013** | **+** | **+** | **1359** | ***Burkholderia metallica*** | **R-16017(T)** | **Vanlaere et al. 2008** | **99.93** |
| ***Bacillus pumilus* strain 104** | **'Valencia' orange** | **2010** | **+** | **+** | **1403** | ***Bacillus pumilus*** | **ATCC 7061(T)** | **Meyer and Gottheil 1901** | **99.93** |
| ***Rhodococcus jialingiae* strain 108** | **'Valencia' orange** | **2010** | **+** | **+** | **1363** | ***Rhodococcus jialingiae*** | **djl-6-2(T)** | **Wang et al. 2010** | **99.93** |

+ indicates presence of halo of inhibition in overlay method.

Supplementary Table 2 Phosphate solubilization and siderophore production traits in rhizospheric bacteria

|  | **Phosphate solubilization (cm*)** | **Siderophore production (cm*)** |
| --- | --- | --- |
| ***Pseudomonas geniculata* strain 95** | **-** | **-** |
| ***Pseudomonas granadensis* strain 100** | **-** | **1.18 ± 0.23** |
| ***Burkholderia territorii* strain A63** | **-** | **-** |
| ***Burkholderia metallica* strain A53** | **1.30 ± 0.02** | **-** |
| ***Bacillus pumilus* strain 104** | **-** |  |
| ***Rhodococcus jialingiae* strain 108** | **-** |  |

*Diameter of halo measured using ImageJ (version 1.49) with standard deviation (n=3).

Supplementary Table 3 *P* value (“dunn test” package) for means separation for the *P. nicotianae* diameter in compartmentalized petri dishes assay.

|  | *B. pumilus* (104) | *R. jialingiae* (108) | *P. geniculata* (95) | *B. territorii* (A63) |
| --- | --- | --- | --- | --- |
| *R. jialingiae* (108) | 0.1425 |  |  |  |
| *P. geniculata* (95) | 0.1447 | 0.4648 |  |  |
| *B. territorii* (A63) | 0.2198 | 0.0372 | 0.0335 |  |
| Control | 0.0116 | 0.0006 | 0.0003 | 0.0775 |

Kruskal-Wallis χ^2^ = 16.8508, df = 4, *P* value = 0.

Supplementary Table 4: Secondary metabolites biosynthesis gene cluster prediction by AntiSMASH.

| **Strain** | **Cluster** | **Type** | **From** | **To** | **Most similar known cluster** |
| --- | --- | --- | --- | --- | --- |
| ***Pseudomonas geniculata* strain 95** | **1** | **Lantipeptide** | **31661** | **61263** | **-** |
|  | **2** | **Lantipeptide** | **69318** | **92035** | **-** |
|  | **3** | **Lassopeptide** | **50889** | **73443** | **-** |
|  | **4** | **NRPS** | **1** | **17903** | **Griseobactin_biosynthetic_gene_cluster (23% of genes show similarity)** |
|  | **5** | **Arylpolyene** | **76759** | **99067** | **APE_Ec_biosynthetic_gene_cluster (36% of genes show similarity)** |
|  | **6** | **Arylpolyene** | **1** | **21276** | **-** |
|  | **7** | **Bacteriocin** | **83161** | **105829** | **Orfamide_biosynthetic_gene_cluster (11% of genes show similarity)** |
|  | **8** | **Microcin** | **1** | **1365** | **-** |
|  | **9** | **Bacteriocin** | **143636** | **154481** | **-** |
| ***Bacillus pumilus* strain 104** | **1** | **NRPS** | **142637** | **226234** | **Lichenysin_biosynthetic_gene_cluster (85% of genes show similarity)** |
|  | **2** | **NRPS -T1pks** | **63941** | **144842** | **Paenilamicin_biosynthetic_gene_cluster (14% of genes show similarity)** |
|  | **3** | **Microcin** | **1** | **4646** | **-** |
|  | **4** | **Bacteriocin** | **389939** | **400265** | **-** |
|  | **5** | **T3pks** | **709780** | **750880** | **-** |
|  | **6** | **Terpene** | **789007** | **810881** | **-** |
|  | **7** | **Siderophore-Terpene** | **1579123** | **1607517** | **Carotenoid_biosynthetic_gene_cluster (33% of genes show similarity)** |
|  | **8** | **Terpene** | **245042** | **265962** | **-** |
|  | **9** | **Other** | **698475** | **739896** | **Bacilysin_biosynthetic_gene_cluster (85% of genes show similarity)** |
|  | **10** | **NRPS** | **953569** | **1003277** | **Bacillibactin_biosynthetic_gene_cluster (53% of genes show similarity)** |
| ***Burkholderia territorii* strain A63** | **1** | **Other** | **27970** | **57696** | **Pyrrolnitrin_biosynthetic_gene_cluster (75% of genes show similarity)** |
|  | **2** | **Bacteriocin** | **39718** | **50533** | **-** |
|  | **3** | **T1pks** | **62948** | **110555** | **-** |
|  | **4** | **Phosphonate** | **62903** | **98429** | **-** |
|  | **5** | **Terpene** | **1** | **21898** | **-** |
|  | **6** | **NRPS** | **1** | **1016** | **-** |
|  | **7** | **NRPS** | **97017** | **137068** | **Pyochelin_biosynthetic_gene_cluster (80% of genes show similarity)** |
|  | **8** | **Terpene** | **1** | **15995** | **-** |
|  | **9** | **Arylpolyene** | **1** | **2250** | **-** |
|  | **10** | **NRPS** | **1** | **11614** | **-** |
|  | **11** | **Terpene** | **1** | **11121** | **-** |
|  | **12** | **Phenazine** | **86375** | **133089** | **Lomofungin_biosynthetic_gene_cluster (34% of genes show similarity)** |
|  | **13** | **Terpene** | **48030** | **68863** | **-** |
|  | **14** | **Terpene** | **6981** | **28009** | **-** |
|  | **15** | **Hserlactone** | **2653** | **17394** | **-** |
|  | **1** | **Terpene** | **42600** | **63598** | **-** |
| ***Burkholderia metallica* strain A53** | **2** | **NRPS** | **1** | **17924** | **-** |
|  | **3** | **Terpene** | **27664** | **49640** | **-** |
|  | **4** | **Terpene** | **7467** | **37850** | **-** |
|  | **5** | **Bacteriocin** | **1** | **5961** | **-** |
|  | **6** | **Other** | **32988** | **75978** | **-** |
|  | **7** | **Terpene** | **31278** | **55382** | **-** |
|  | **8** | **Ectoine** | **3735** | **14133** | **-** |
|  | **9** | **Terpene** | **13100** | **33933** | **-** |
|  | **10** | **Terpene** | **1** | **11107** | **-** |
|  | **11** | **Other** | **2018** | **34354** | **Pyrrolnitrin_biosynthetic_gene_cluster (100% of genes show similarity)** |
|  | **12** | **Terpene** | **14830** | **37505** | **-** |
|  | **13** | **Phosphonate** | **1** | **36125** | **-** |
|  | **14** | **Hserlactone** | **36730** | **54805** | **-** |
|  | **15** | **T1pks** | **43661** | **91265** | **Lipopolysaccharide_biosynthetic_gene_cluster (5% of genes show similarity)** |

# Supplementary Materials

The antagonism assay by overlay method was performed for *Colletotrichum acutatum*, *Phyllosticta citricarpa* and *Phytophthora nicotianae*. NA plates were inoculated with 1 μL of bacterial suspension (10^8^ cfu/mL) and grown for three days at 28˚C. Petri dishes were then subject to chloroform vapor for a few hours until completely evaporated. The appropriate medium was used for each isolate in soft agar (0.06%) at 10^6^ spores/mL.

*C. acutatum* spores were obtained by flooding one week old cultures with 3 mL of sterile distilled water and gently removing the surface of the petri dish with a sterile spreader. The resulting suspension was then filtered using three layers of cheesecloth. *Phyllosticta citricarpa* mycelial fragments were collected as described previously (Hincapie et al., 2013). *Phytophthora nicotianae* zoospores were obtained by culturing four mycelium plugs of the isolate on V8 broth in the dark. After one week medium was replaced with sterile distilled water and left growing under dark/light conditions. To induce zoospore release, *Phytophthora nicotianae* was cooled at 4 °C for one hour and then kept at room temperature for one hour. Spore concentration was determined using Neubauer hemocytometer and was adjusted accordingly to obtain a final concentration of 10^6^ spores/mL.

**3 Supplementary Figures**

**Supplementary Figure 1.** Overlay antimicrobial activity in vitro. A. *Burkholderia* isolates have antifungal activity against *Colletotrichum acutatum*. B. The antifungal activities of *Burkholderia metallica* strain A53 and *Bacillus pumilus* strain 104 against *Phyllosticta citricarpa* mycelial

fragments. C. *Burkholderia* strains and *Bacillus pumilus* inhibition activity against zoospores of *Phytophthora nicotianae* in vitro.

**Supplementary Figure 2.** Heat map representing the number of genes involved in osmoprotection. Enzymes involved in the synthesis of trehalose and cardiolipin are shown for all four bacteria.

**Supplementary Figure 3.** Butanoate metabolism (KEGG). *B. pumilus* strain 104 harbors the gene for 2,3-butanediol dehydrogenase (EC 1.1.1.4) that catalyses the production of 2,3 butanediol from 2-acetoin and the reverse reaction as well as the gene encoding alpha-acetolactate decarboxylase (EC 4.1.1.5) involved in the synthesis of 2-acetoin from acetolactate and the gene that encodes the acetolactate synthase small subunit (EC 2.2.1.6) that produces 2-acetolactate from pyruvate.

**Supplementary Figure 4.** Structural prediction of the NRPS antibiotic encoded in *B. pumilus* strain 104 Cluster 1. AntiSMASH software identified a putative NRPS antimicrobial biosynthesis cluster. Based on the domain organization, the prediction of the putative structure is shown.
